# Plasmapheresis in a patient with rhabdomyolysis: a case report

**DOI:** 10.4076/1757-1626-2-8138

**Published:** 2009-08-12

**Authors:** Rohina Swaroop, Raja Zabaneh, Nakul Parimoo

**Affiliations:** Northwest Louisiana Nephrology1800 Buckner Street, Suite C-120, Shreveport, LA 71101USA

## Abstract

**Introduction:**

Cardiovascular benefits and improved survival have resulted in statins becoming the most prescribed drugs in USA. There is a small but significant risk of developing statin induced rhabdomyolysis especially in combination with other lipid lowering medications for example fibrates like Gemfibrozil.

**Case presentation:**

We describe a case of an 82 year old male patient who developed rhabdomyolysis while taking a combination of Simvastatin and Gemfibrozil and was successfully managed with plasmapheresis.

**Conclusion:**

There are only a few cases of rhabdomyolysis treated with plasmapheresis reported in literature. Most of the case reports of using this technique in rhabdomyolysis have been from Asia, Australia and Europe. We suggest that plasmapheresis may be considered as an option in severe life threatening rhabdomyolysis.

## Introduction

Acute rhabdomyolysis is a syndrome characterized by the breakdown of skeletal muscle resulting in subsequent release of intracellular contents into the circulatory system, which can cause potentially lethal complications. These contents include myoglobin, potassium, aldolase, lactate dehydrogenase, creatine phosphokinase and glutamic oxaloacetic transaminase.

 Acute renal failure is one of the most common complications of rhabdomyolysis. HMG-CoA reductase inhibitors are the most commonly prescribed lipid lowering medications. Induced deficiency of Co-enzyme Q, a mitochondrial and lipid membrane antioxidant and a cofactor in mitochondrial ATP production has been proposed as the possible cause for statin related muscle injury.

The traditional management of rhabdomyolysis induced acute renal failure includes aggressive intravenous fluid resuscitation, use of mannitol and bicarbonate is also notable for reducing risk of ARF. However evidence has not really proven benefit of these measures over use of intravenous fluid administration. Standard hemodialysis techniques have limited ability to remove circulating myoglobin (proposed reasons for this, see under Discussion).

There is a paucity of literature regarding use of plasmapheresis in rhabdomyolysis. The available literature is detailed/ reviewed in the Discussion section of this article.

## Case presentation

An 82 year old white male was admitted with 10 day history of severe, continuous myalgia and proximal muscle weakness. He denied vigorous physical exercise, heat exposure, alcohol or recreational drug abuse. He reports no other specific complaints.

His medications Simvastatin 80 mg qhs, Metoprolol extended release 25 mg once daily, Hydrocodone/Acetaminophen 7.5/500 mg p.r.n. for pain, cyclobenzaprine 10 mg t.i.d. p.r.n. for muscle spasms and lisinopril 10 mg once daily. He had been on simvastatin for many years, pertinent to note that Gemfibrozil 600 mg b.i.d. had been started a month ago. He reports no significant food or drug allergies.

Past medical/surgical history of viral cardiomyopathy 5 years back, migraines, obstructive sleep apnea, shoulder and foot surgery, appendecectomy, Cervical and lumbosacral spondylosis status post laminectomy , BPH status post transurethral resection of prostate and non melanotic skin cancer. Family history significant for hypertension.

On admission serum K 5.9, Na 132, BUN 73, Cr 2.2, albumin 2.7, chloride 98, CO2 21, CBC was within normal limits (Table 1) CK was 41368 on presentation (n=35-232 U/L), ALT was 1293 (n=30-65U/L) AST 1439 (n=15-37) phosphorus 5.4, calcium 9.5. UA showed 3+ occult blood with only 4-10 RBCs per high power field.

**Table 1.** Patient’s laboratory values from day 1 to day 30

Day	Creatinine Kinase	BUN	SGPT(ALT)	SGOT(AST)	Albumin	Calcium	Creatinine	Potassium
1	41368	73	1293	1439	2.7	9.5	2.2	5.9
2	26477	70	1441	1660	2.3	8.7	2.2	5.2
3	45504	62	1585	1842	2.1	8.3	1.8	3.8
4*	86400	82	930	1326	3.3	7.2	2.6	3.6
5	85578	105	983	996	2.9	5.5	2.3	4.1
6	29698	56	839	769	2.9	6.2	2.4	3.8
7	27291	76	610	410	2.6	6.6	3.6	4.0
8**	10330	70	765	339	2.5	8.1	4.2	4.7
15	131	60	188	34	3.2	9.1	3.6	3.6
30	96	14	13	17	3.4	8.9	1.9	4.4

Red denotes higher than normal and blue denotes lower than normal values.

*plasmapheresis initiated, **plasmapheresis discontinued.

Acute renal failure and abnormal liver function tests secondary to rhabdomyolysis was diagnosed. Simvastatin and Gemfibrozil were stopped and he was started on aggressive alkaline intravenous fluid resuscitation. In light of the elevated potassium and ARF, ACE-I was also stopped.

By hospital day 4 creatinine kinase rose to 86400, BUN 82, Cr 2, ALT 930 and AST 1326. UA showed 3+occult blood with no red blood cells on microscopic examination. He became oliguric; plasmapheresis with one and a half volume replacement with albumin was started at this point in addition to hemodialysis. The patient received plasmapheresis daily for 5 days in addition to hemodialysis

Patient was discharged to Physical Medicine and Rehabilitation at Day 30, he remained off dialysis and plasmapheresis and was discharged in stable ambulatory status.

Approximately 6 months after this episode all laboratory parameters were within normal limits, he remained at baseline health as well as functional status. Approximately 4 years after this episode, at his last outpatient nephrology clinic visit all his laboratory values remain within normal limits and he maintains baseline health and functional status.

Omega-3 fish oil supplements, diet and lifestyle changes were used to treat his abnormal lipid profile, statins and fibrates were avoided due to this episode and also due to patient’s desire that he not be treated with either of the two medications. It is pertinent to note that his last lipid profile meets ATP III guideline goals.

## Discussion

Acute rhabdomyolysis is characterized by the breakdown of skeletal muscles resulting in subsequent release of intracellular contents into the circulatory system, which can cause potentially lethal complications. These contents include myoglobin, potassium, aldolase, lactate dehydrogenase, creatine phosphokinase and glutamic -oxaloacetic transaminase.

A potentially lethal complication of rhabdomyolysis is acute renal failure. 3 hydroxy-3- methyl glutaryl coenzyme A (HMG -CoA) reductase inhibitors are among the most commonly prescribed drugs in the USA. Upto 5% of patients taking statins may have muscle related complaints, typically myalgias; however myositis and rhabdomyolysis do occur.

Induced deficiency of Co-enzyme Q which is a mitochondrial and lipid membrane antioxidant and cofactor in mitochondrial ATP production has been proposed as a possible cause for statin related muscle injury.

When combined with gemfibrozil, all statins except fluvastatin have been associated with reports of rhabdomyolysis, it is pertinent to this case that gemfibrozil was added to simvastatin resulting in rhabdomyolysis [1-3]. Although the reason for the increased risk of rhabdomyolysis with combination therapy is not clear, a possible explanation is that gemfibrozil increases serum concentration of active statin acids, the risk of statin induced myopathy is enhanced in the elderly [3], note that this patient is 82 years old. Risk of ARF after rhabdomyolysis has been estimated to be between 17 % and 35 % [4,5].

Management of myoglobinuric acute renal failure includes aggressive intravenous fluid (typically alkaline) administration to restore the hypovolemia. While mannitol and bicarbonate are considered standard care in preventing acute renal failure in these patients the available evidence suggests that that there is no benefit over and above aggressive fluid resuscitation [5,6].

The techniques and devices used for classic dialytic technique have displayed a limited capacity for the removal of circulating myoglobin [6]; myoglobin’s non-spherical shape and electrical charge make it a solute with an Einstein-Stokes radius greater than expected. This results in low diffusion coefficient, requiring transport by convection; myoglobin also possesses a steric magnitude that is likely to be rejected by most high flux dialyzer membrane pores.

Literature containing use of plasmapheresis in rhabdomyolysis is scarce and anecdotal.

Ronco [6] reported that attempts to use plasmapheresis in myoglobinemia have resulted in higher sieving coefficients, but notes limitations due to low volume exchanges.

Kuroda et al [7] reported successful treatment by plasma exchange of acute renal failure, DIC and multiorgan damage associated with extensive muscle damage.

Paaske et al [8] reports on a patient with acute compartment syndrome causing rhabdomyolysis, ARF, and was successfully managed with plasmapheresis.

Cornellisen et al [9] evaluated 4 patients with rhabdomyolysis. The results showed that a single 2 liter plasma exchange has no beneficial effects in the treatment of patients with rhabdomyolysis. It is prudent to note only one plasma exchange was done; all other case reports have used multiple daily exchanges.

Yang et al [10] used plasma exchange in a case of rhabdomyolysis complicated with increased serum Bezafibrate level .They advocated that Bezafibrate being highly protein bound is unlikely to be cleared by hemodialysis .They suggested that plasma exchange is safe and effective in addition to supportive care for rhabdomyolysis associated with excessive Bezafibrate.

## Conclusion

Rhabdomyolysis is a potentially life threatening condition that should be suspected in all patients on statins presenting with myalgias. Patients on combination therapy with other medications like Gemfibrozil have a higher risk of developing it.

We learn from this case that physicians should be vigilant regarding this potentially lethal adverse effect, laboratory follow up should be maintained on liver function and muscle enzyme levels. Physicians must ensure that the patient is appropriately educated about potential adverse reactions including rhabdomyolysis before prescribing these medications. A well informed patient can notify his physician in a timely fashion, thereby potentially avoiding a life-threatening complication.

In this case especially keeping in mind the steady reduction in toxic muscle enzyme levels with use of plasmapheresis , we recommend that plasma exchange be considered in cases of severe rhabdomyolysis and acute renal failure not responding to the standard medical management of intravenous fluid resuscitation.

## Abbreviations

ACE-I: angiotensin converting enzyme inhibitors
ALT: alanine amino transferase
ARF: acute renal failure
AST: aspartate aminotransferase
BUN: blood urea nitrogen
CBC: complete blood count
CK: creatine kinase
CO_2_: carbon dioxide
Cr: creatinine
HMG -CoA: 3 hydroxy-3 methyl-glutaryl-coenzyme A
K: potassium
Na: sodium
RBC: red blood corpuscles
UA: urine analysis
